# Benefits—Dental and Otherwise

**Published:** 1888-11

**Authors:** C. C. Barker

**Affiliations:** Meriden, Conn.


### BENEFITS—DENTAL AND OTHERWISE.

BY C. C. BARKER, MERIDEN, CONN.

READ BEFORE THE CONNECTICUT VALLEY AND MASSACHUSETTS DENTAL SOCIETIES, BOSTON JULY 10, 1888.

The dental practitioners, who in this year of grace, 1888, fulfills the duties of his chosen specialty, has at hand many advantages which come to him like a blessing, and deserve the name of *benefits*—benefits resultant from the efforts, investigations and labors of others—pioneers, predecessors and co-laborers, which demand and deserve our recognition and appreciation.

You who have noticed the derivation of the term anatomy, (*ana*, " up," and *temnein*," to cut") will remember that it originally signified a cutting ; a " dissection of organized bodies so as to expose the structure, situation and use of the various parts."

If we accept and insist upon this primal sense, then we—dentists—are most certainly and practically *anatomists;* for our life-work, day by day, week by week, month by month, and year by year, is a cutting—a dissection—the everlasting cutting of the dental and oral tissues.

But the term in accordance with modern usage " has been appropriated," says Dunglison, " to the study and knowledge of the number, shape, situation, structure and connection, in a word, of all the apparent properties of organized bodies."

Very properly and wisely, those in charge of our dental schools have made anatomy and physiology fundamentals in the curriculum.

And it is not easy to overestimate the importance of these studies as foundations upon which to base an intelligent and successful practice in our specialty. Modern dentistry, with its wonderful achievements and blessings, is thus based ; and the benefits which the world has received at its hand, doubtless began with, and are co-incident with the special study of the dental organs.

With the first crude knowledge came the idea and commencement of efforts at conservation ; and the successes gained, and benefits attained since that time, run in almost parallel line with the advance of knowledge in this regard.

I considered these facts as axiomatic and self-apparent.

A knowledge of structure and organization, or anatomy, is necessary in order that we understand *function* and vital phenomena, which we call physiology.

We must recognize true physiological action that we may properly distinguish the expression of disease, perverted action, and which latter we call pathology.

Pathological perception is required that we may appropriately treat the diseased condition either for its removal or alleviation; the treatment which we technically denominate as therapeutics.

I make this simple statement of sequence to more plainly emphasize the fact already insisted upon that our dental knowledge finds its true foundation in anatomy and physiology.

I cannot attempt to elaborate or to enumerate in any detailed manner the benefits derived since the birth of this knowledge. I shall only aim to speak suggestively in a general way, and later on of some specific points of more definite interest to ourselves as practitioners.

Dental knowledge, like that in many another department, is of comparatively recent origin.

Following the discovery of enamel and its function came the idea of filling, or "stopping" a tooth, as the operation was then called.

It was so like stopping a leak—The very term shows that the thing was done with some philosophical sense.

I need not trace accurately the history through all the bitter experience during which it became plain that a filling cannot with impunity impinge upon or interfere with the function of the pulp, etc., etc.

It is all a matter of intellectual development, and benefits accruing therefrom; to which each one of you can give special testimony.

How great the advance since that first dawning light in knowledge and in practice up to the present. When now, through a recognition of the vitality of the cementum, and the almost vicarious action of the peridental membrane, many an almost depopulated mouth can be rehabilitated with a worthy denture, rooted and grounded upon nature's own foundation; and many a root which had hitherto dwelt in great tribulation happily wears a golden crown.

Is the alleviation and cure of dental irritation, and the prolongation of human life in comfortable and happy circumstances a benefit?

Is it anything to preserve or supply to man his masticatory apparatus, whereby the nutritive functions continue unimpaired, and "by reason of strength" man attains unto his four score years or more?

These are the blessings which modern dentistry confers upon human kind, everywhere pertinent and grand.

And right here, in passing, I wish personally to express my humble acknowledgments to the pioneers and workers of the past and present, through whose devoted and toilsome efforts the present accretion of knowledge has been attained.

We dentists—whether we are conscious of it or not—are enjoying the fruit of their labors. It is a question—not altogether out of order—whether we properly sense the debt of gratitude we owe. May I not suggest that if it has not been already done, these dental societies of New England should lose no time in placing themselves on record in acknowledgment of the service rendered to our specialty by the co-laborers of the past and present.

Their efforts, however humble some may consider them, have been stepping-stones and vantage ground for farther progress. Do you speak slightingly of some of their methods as crude and untenable? So will some of those who are to come speak of us to-morrow. Even their blunders confer benefits upon us, as ours certainly will upon the new generation, provided they have wisdom enough to profit by our experience.

It is not many years, really, since dentists began practically to magnify their calling; and the results have proved that those structures upon which we are called to expend our energies and skill will bear magnifying. The greatest advance yet made in knowledge of the dental tissues has been wrought by the aid of the microscope.

There is a little band of skilled workers in our ranks. All honor to them for their patience, assiduity and untiring devotion. They have done us great service. Not all of us are microscopists; indeed, only a few are.

It requires constitutional fitness to use the binocular well; and time and opportunity, and the very best eyesight, aided by a trusty objective. These things we cannot all furnish. One essential element of constitutional fitness, I apprehend to be a calm and accurate judgment of form, unswerved by exuberance of imagination.

You know one man, looking overhead in the night, discerns fantastic shapes in the starry heavens; while his companion at his side, equally intelligent, sees nothing of the sort. Some claim to often hear whisperings from the angels; some claim frequent temptations from the devil; while others still never hear anything from either angel or devil.

Imagination creates appearances, and gives more color to them than the rainbow itself can furnish.

The eye, without the assistance of a powerful lens, fails to discern the complex structure of the teeth, a structure which the microscope has proven to be *sui generis.*

By its means the startling discovery was made that the teeth are developed in the mucous membrane, and are not, in proper reality, members of the bony skeleton; but, instead, parts of the "outer or dermal system," and of similar origin as the nails and hair.

The principles of dental embryology and histology are doubtless well and correctly established in the main, and are clearly and beautifully presented to us in the writings and illustrations given by Dr. Sudduth of Philadelphia, Dr. Andrews of Cambridge—our honored President, Dr. Stowell of Ann Arbor, Drs. Heitzman, Abbott and Bœdecker of New York, and others.

Although differing somewhat in detail, with here and there a "missing link"—proof that some at least, if not all of the brethren, still "see through a glass darkly." Yet, notwithstanding this, the histological character, nature and function of the tissue elements composing the dental structures has been so well apprehended and revealed as to greatly aid our conception of proper hygienic and therapeutic treatment; and we and our patients have been correspondingly benefited.

But too much must not be expected from the microscope.

There are those who talk as if they believed that most problems will yet be solved by the medium of the all-powerful lens.

I do not for a moment believe it.

Proof that this *cannot be* is very readily furnished.

For instance, protoplasm is the unit from which all our beginnings proceed. It is what the etymology of its name signifies—protos—first, and plasso—to form—that is—the first form of living matter.

We know that the life of every animal organism begins in the ovarian egg, which is declared by embryologists to be "a minute globule of protoplasm." They also state emphatically that "the undeveloped ovarian egg, immediately after its fertilization, is uniform in appearance throughout the animal kingdom, the human ovum at this stage corresponding in structure to those which stand at the very foot of the zoological scale."

Now, who cannot fail to realize, with a moment's reflection, that in this immense ovarian group, from which over one hundred thousand distinct species of animal life are evolved, that each one of those eggs, which, remember, as nearly as can be discerned with the most powerful lens, are only "minute globules of protoplasm," and "uniform in appearance." Yet each one of them represents a distinct species, differing from each other in almost infinite variations.

This one will be a pig; that, an elephant; this, a frog; that, a man.

It's a long catalogue—over 100,000 names—and all varying to an indescribable degree.

But here in the beginning all are alike, so far as human ability employing every aid can distinguish, and we call them protoplasm, which conveys to us the sense of homogeneousness, likeness and simplicity of nature.

But their subsequent varying development proves that this likeness in the early stage is a *seeming*, rather than real; for who can doubt that wrapped up in these protoplasmic globules, we have the distinct plan, the *prevision* of the creature that is to be. So what we call proto—first—is not first after all.

No. 1 lies further back.

There is something resident in these little granules which escapes our keenest visual penetration, and eludes one test of our finest chemistry. So you see on two sides, both the Physicist and the Chemist reach impassible barriers in their attempts at ultimate analysis.

I have spoken thus for the variations between species; allow me to call your thoughts to the differences which distinguish members of a single group from each other. We will take our own group—the human mammal—man.

How varied are the combinations of organs, as in the sexes—the differences in size, form, weight, color, texture, relative development of parts; in short, all those distinctive features which give peculiarity and individuality to every single member of the human family, all these marks reside in that original bit of protoplasm.

And this is not all; neither can I attempt to tell all; but that which determines the mental development, the grade and character of the intellect, the temperament, diathesis and every idiosyncrasy to which humanity is heir, *is there* wrapped up in that little cell.

And of course we shall not fail to remember that the potentiality and the detail of plan which shall weave the teeth themselves, according to a definite character of form and texture, and attribute, is resident also in this marvellous atom, which after all is no atom at all.

Do I believe in heredity?

Most certainly, and not in a loose, general way, but in the finest detail.

I suppose no argument is necessary here to establish the foundation of this truth.

A recent writer correctly states that "No fact is more pregnant with awful meaning than the facts of the inheritance of disease.

It meets the physician on his daily rounds, paralyzing his art and filling him with sadness.

The legend of the ancient Greeks pictured the malignant Furies pursuing families from generation to generation and rendering them desolate.

The furies still ply their work of terror and death; but we have stripped them of the garb which superstition threw around them, and they now appear to our eyes in the more intelligible but not less awful form of hereditary disease.

Modern science, which has cast illumination into so many dark corners of nature, has shed a new and still more lurid light on the words of the Hebrew scripture: "The sins of the father shall be visited upon the children unto the third and fourth generation."

Sir Henry Holland says that "No organ or texture of the body is exempt from the chance of being the subject of hereditary disease." Our own experience as dentists furnishes us daily proof that the teeth must be included in the category.

And when I say this, I mean to go so far as to state that every tooth in the human mouth has an individuality of form and size, and color and texture of position and articulation which in the larger degree has been determined by the parentage.

Positive proofs of this are attainable wherever we have the opportunity to carefully compare the conformation of the dental arches of parents and their children, and notice how the teeth of the younger generation seem in so many instances the copy of those in the mouths of the older, which stand there like original types. A most striking instance has occurred in my own practice within the last year.

These marked examples are only the more striking and apparent instances of that law of heredity which determines the character of the dental structures.

Every practitioner within even limited experience, quickly discovers that there are many grades of tooth texture—from that of almost chalky softness to that of nearly flint-like hardness—with corresponding capacity and endurance.

Of these variations, the microscope has as yet failed to give us anything like an intelligible or recognized classification, or to emphasize to us what we may consider as the ideal tooth.

But the dental surgeon, as he cuts with the excavator, burr, or drill, is immediately aware of these varieties; not always through the medium of sight, but by virtue of the sense of feeling, of touch, and as we well know by means of this recognition which comes to us primally through the touch—the feel. Sight itself becomes educated to that degree that unaided by any microscope, it can often identify these varying tooth characters without the contact of touch.

There are some teeth so exceedingly frail in point of structure, that the finest operations within the capacity of dental skill will fail to save; and the hardest thing perhaps for patients to understand is this variation in tooth character and structure.

Until they are able to discern this more fully, so long will they fail to distinguish carefully between good operations and poor.

